# Restoration of Vegetation in Relation to Soil Properties of Spoil Heap Heavily Contaminated with Heavy Metals

**DOI:** 10.1007/s11270-018-4040-6

**Published:** 2018-11-24

**Authors:** Marek Pająk, Ewa Błońska, Marta Szostak, Michał Gąsiorek, Marcin Pietrzykowski, Otmar Urban, Piotr Derbis

**Affiliations:** 1 Department of Forest Ecology and Reclamation, University of Agriculture in Krakow, al. 29 Listopada 46, 31-425 Krakow, Poland; 2 Department of Forest Soil, University of Agriculture in Krakow, al. 29 Listopada 46, 31-425 Krakow, Poland; 3 Department of Forest Management, Geomatics and Forest Economics, University of Agriculture in Krakow, al. 29 Listopada 46, 31-425 Krakow, Poland; 4 Department of Soil Science and Soil Protection, University of Agriculture in Krakow, al. Mickiewicza 21, 31-120 Krakow, Poland; 5 Global Change Research Institute, Czech Academy Sciences, Bělidla 986/4a, 60300 Brno, Czech Republic

**Keywords:** Soil contamination, Microbial activity, Phytoremediation, *Silene vulgaris*, *Pinus sylvestris*

## Abstract

The main objectives of our study were to evaluate soil contamination on a zinc-lead spoil heap in the Upper Silesian Industrial Region in southern Poland using pollution indices, and to investigate the relation between soil properties and the natural succession of vegetation. Organic carbon and nitrogen, pH, soil texture, base cations, and heavy metal content were analyzed in soil samples at depths of 0–15 cm below the organic horizon over a regular grid of 14 sampling plots. The contents of Zn, Pb, and Cd exceeded by several times the acceptable thresholds. Measurements of soil enzyme activity were used to evaluate the progress of vegetation development in relation to soil chemical properties. The results indicate that heavy metals had a significant impact on soil enzyme activity and the development of vegetation cover. High contents of Pb and Cd reduced enzyme activity, while this activity increased with increasing amounts of soil organic matter. Further, the accumulative capacities of heavy metals in needles of Scots pine (*Pinus sylvestris* L.) and aboveground biomass of bladder campion (*Silene vulgaris* (Moench) Garcke) were examined. A high accumulation of Zn, Pb, and Cd in the aboveground tissues of *S. vulgaris* indicated an unusual tolerance of this species to heavy metals and the possibility of using this species in phytoremediation of post-industrial sites.

## Introduction

Industrial activity related to extraction and processing of zinc and lead ore resources has had a major impact on the natural environment and particularly soil conditions (Krzaklewski and Pietrzykowski 2002; Krzaklewski et al. 2004). A high content of heavy metals in soils, waters, and sediments are often observed near mine or ore processing sites (Li et al. 2001; Krzaklewski et al. 2004; Pająk et al. 2016; Pająk et al. 2017). Numerous studies have shown that heavy metals are extremely persistent in the environment and not biodegradable (Grzebisz et al. 2002; Moosavi and Zarasvandi 2009). Spoil heaps resulting from technological processes are often characterized by unique chemical and physical properties and especially by high contents of heavy metals (Krzaklewski and Pietrzykowski 2002; Matini et al. 2011).

The total content of heavy metals in such soils is related to the accumulative capacity of soils, size of the soil organic fraction, and/or soil pH (Šmejkalová et al. 2003; Ming-kui and Calvert 2005, Błońska et al. 2016a; Gąsiorek et al. 2017). In particular, soil organic matter substantially influences the chelation of heavy metals. Weber et al. (2018) reported that soil organic matter stimulates retention, mobilization, and migration of heavy metals in soil. Stable, insoluble organic matter rich in high-molecular mass humic acids plays an important role in heavy metal immobilization (Kabata-Pendias 2011).

High amounts of heavy metals in the environment are toxic for most organisms and thus limit an acclimation of vegetation and natural succession in contaminated areas (Remon et al. 2005, Szarek-Łukaszewska and Grodzińska 2007). Only few species of herbaceous plants and trees have shown a high tolerance to such unfavorable environmental conditions (Ciarkowska et al. 2016, Gezer and Cooper 2016).

Heavy metals strongly affect the biological properties of soils. Numerous studies have reported a reduced activity of enzymes involved in C and N cycling in soils with increasing heavy metal content (Ekenler and Tabatabai 2002; Gao et al. 2010; Ciarkowska et al. 2014). Due to the known long-term impact of heavy metals on microorganisms, their growth and metabolism are likely to be negatively affected (Kandeler et al. 2000; Qu et al. 2011). The activity of soil enzymes is a sensitive indicator of soil quality (Wang et al. 2007) and is proposed accordingly as a reliable tool to monitor changes in soils (Klamerus-Iwan et al. 2015).

Amongst the numerous soil enzymes, dehydrogenases, β-glucosidase, and urease have shown exceptional significance for assessing soil condition. The activity of dehydrogenases is directly associated with soil organic matter decomposition (Margesin et al. 2000), and it has been used as a soil quality indicator (Rodriguez et al. 2004; Błońska et al. 2016b). Urease, occurring in many vascular plants and microorganisms (Qu et al. 2011), catalyzes hydrolytic decomposition of urea into carbon dioxide and ammonia and is released during the process of mineralization of organic matter. It has been shown that reduced urease activity is an effective marker of soil contamination by Cd, Zn, Pb, and/or PAH (Shen et al. 2005). The remaining indicator, β-glucosidase, is a key microbial enzyme involved in carbon cycle released during plant litter decomposition (Sinsabaugh et al. 1991).

Besides an absolute content of heavy metals, anthropogenic soil pollution can be assessed by numerous soil pollution indices. These indices allow a complex evaluation of soil quality, environmental risk, and soil degradation (Mazurek et al. 2017) and thus may be more appropriate for the assessment of heavy metal contamination (Kowalska et al. 2016). The accumulation of heavy metals in soils may have various sources; therefore, it is necessary to use an appropriate local or reference background to correctly interpret the calculated soil pollution indices. The most commonly used reference backgrounds are the elemental content of continental crust or surface soils as reported by Rudnick and Gao (2003) and Kabata-Pendias (2011). The use of various geochemical backgrounds allows a comprehensive and objective assessment of soil pollution level (Kowalska et al. 2016; Gąsiorek et al. 2017; Mazurek et al. 2017).

It has been shown that some plant species are capable of accumulating substantial amounts of heavy metals in their tissues (Monfared et al. 2013; Pietrzykowski et al. 2014; Zhou et al. 2016; Pająk et al. 2017). To quantify the ability of plant tissues, particularly foliar tissues, to accumulate heavy metals, a metal accumulation index (MAI) has been developed (Liu et al. 2007).

Our study aimed to assess the pollution of soils forming on a zinc and lead ore–processing residue heap. The assessment of heavy metal pollution was based on an evaluation using soil pollution indices as well as an assessment of the accumulation of selected trace elements in needles of Scots pine (*Pinus sylvestris* L.) and the aboveground biomass of *Silene vulgaris* (Moench) Garcke) (bladder campion). The choice of these species was dictated by the natural succession on all studied plots. Moreover, a relation between soil properties, reflected particularly by enzymatic activity and the natural succession of vegetation was also investigated. The following hypotheses were tested: 1) the soils forming the spoil heap are strongly polluted with heavy metals, leading to high values of soil pollution indices; 2) the natural vegetation succession is determined by the content of heavy metals and 3) it is significantly associated with enzyme activity in the spoil heap soils; and 4) the herbaceous plant *S. vulgaris* accumulates higher amounts of heavy metals than coniferous *P. sylvestris* and thus may be a promising candidate for phytoremediation at post-industrial sites.

## Materials and Methods

### Study Area

**Fig. 1:**
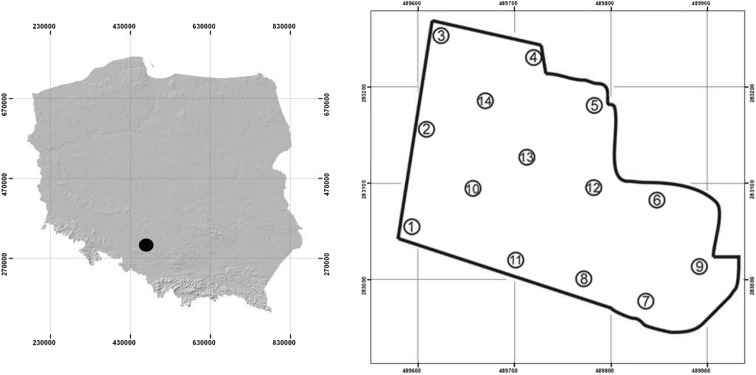
Location of the research area (Upper Silesian Industrial Region, southern Poland) (left) and location of research plots on the site (right)

**Fig. 2:**
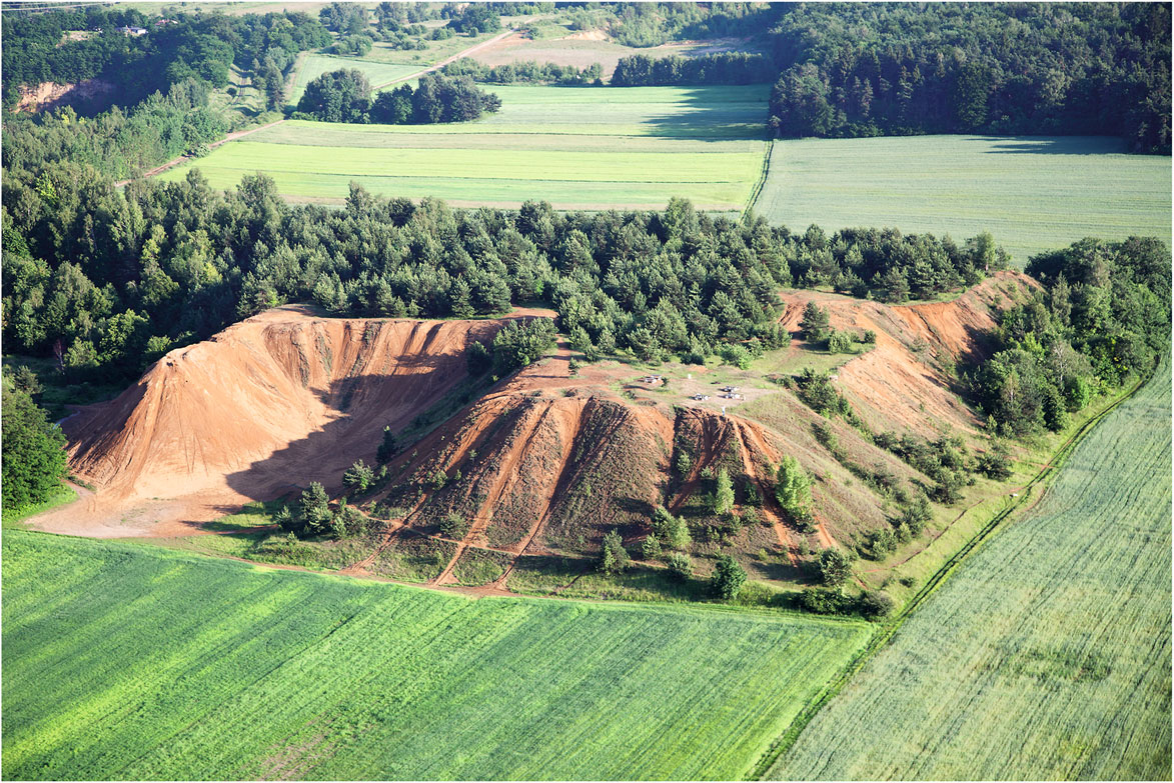
Aerial view of the spoil heap (photo Romankiewicz 2012)

The study was carried out in August 2011 on a spoil heap consisting of processed material left after rinsing of zinc and lead ores in the “Fryderyk” mine in Tarnowskie Góry (50° 24′ 54″ N, 18° 51′ 17″ E; Upper Silesian Industrial Region, southern Poland) (Fig. 1, left; Fig. 2). The formation of the spoil heap began in 1840 and ended in 1912. The waste dump takes up an area of over 6 ha. The shape of spoil heap is irregular, with a flat top and steep slopes of 35–45°. The height of the heap in the south-eastern part is 10–15 m, increasing to ca. 17 m in its western part, and reaches its highest value in the northern part of the heap, namely 22 m above the level of the surrounding terrain. The spoil heap emerged as a result of accumulation of waste material, i.e., is mainly composed of chunks of metal-bearing dolomite, remains of crushing and rinsing of galena, followed by calamine and limonite. The study site has not been subject to any reclamation treatments.

To detect spatial heterogeneity, the spoil heap surface was covered with a regular sampling network of 14 plots (Fig. 1, right). The area of each plot was 100 m^2^. Five soil samples were taken at each plot, each from a depth of 0–15 cm below the organic horizon, to form a composite sample.

For determining the level of accumulation of the selected trace elements (Zn, Pb, Cd, Mn, Cr, Cu, and Ni) in vegetation cover, sprouts (with second-generation needles) from five Scots pine trees per trial plot were sampled. The second-generation needles were isolated from sprouts were obtained from the middle part of the tree crowns on their south-eastern side. In addition, the aboveground biomass (leaves and stems) of the metallophile *S. vulgaris* was collected in all investigated areas. In order to assess plant succession, historical aerial photos from 1947 to 2011 were also used.

### Chemical Analyses

The soil samples were dried and then sieved through a sieve with a mesh diameter of 2 mm. For every sample, chemical properties were determined over two replications. For the determination of soil pH, the potentiometric method was used; soil pH was measured in a 1:2.5 (*w*/*v*) suspension of distilled water and 1 M KCl. The soil texture (sand diameter 2–0.05 mm, silt diameter 0.05–0.002 mm, clay diameter < 0.002 mm) was determined with the use of sieve method combined with the aerometric method according to the PN-R-04032 (1998). The content of soil total organic carbon (TOC) and total nitrogen (N_t_) was measured with a LECO CNS 2000 elemental analyzer. Inductively coupled plasma optical emission spectrometry (ICP-OES) was applied to determine the concentration of exchangeable cations using an iCAP 6500 DUO spectrometer (Thermo Fisher Scientific, Cambridge, UK) and the sum of base cations (BC) was subsequently calculated. The same technique and apparatus was used to determine the content of investigated elements (Fe, Zn, Pb, Cd, Mn, Cr, Cu, and Ni) after prior mineralization of soil samples using a mixture of concentrated nitric and perchloric acids at a ratio of 2:1 (Carter and Gregorich 2006).

Dried samples of Scots pine needles and aboveground fragments of *S. vulgaris* were homogenized using a knife mill GM 200 (Retsch, Haan, Germany) and subsequently mineralized in a mixture of HNO_3_ and HClO_4_ (3:1). An ICP-OES emission spectrometer (Thermo Fisher Scientific, UK) was used to determine the contents of the investigated trace elements. The accuracy of analytical results was checked by a parallel analysis of a certified European reference plant material (ERM-CD281; rye grass).

### Enzyme Analysis

For the determination of soil enzymatic activity, fresh soil samples having their natural moisture content (ranging between 8 and 14.5%) were sieved through a sieve (ø 2 mm) and stored at a temperature of 4 °C before the analysis was performed. The activities of dehydrogenase (EC 1.1.1.1), urease (EC 3.5.1.5), and β-glucosidase (EC 3.2.1.21) were determined in three repetitions. Dehydrogenase activity (DH) was established by the reduction of 2,3,5-triphenyltetrazolium chloride (TTC) to triphenyl formazan (TPF) using Lenhard’s method according to the Casida procedure (Alef and Nannipieri, 1995). Briefly, 6 g of soil was incubated with 1 ml of 3% TTC for 24 h at a temperature of 37 °C. The TPF produced was extracted with ethyl alcohol (95%) that was contaminated with methanol. TPF was measured spectrophotometrically (UV-Vis, Cary 300, Varian, USA) at 485 nm. Urease activity (UR) was determined according to Tabatabai and Bremner (1972), with a water urea solution as a substrate. This activity was determined by the amount of NH_4_^+^ released after incubating the sample for 2 h at a temperature of 37 °C. The concentration of NH_4_^+^ was measured at 410 nm by means of the colorimetric method (Alef and Nannipieri, 1995). The activity of β-glucosidase (BG) was determined employing the method developed by Eivazi and Tabatabai (1988) with later modifications (Alef and Nannipieri 1995), using p-nitrophenyl-β-*D*-glucopyranoside (PNG) as a substrate.

### Determination of Vegetation Parameters

In each sampling plot, the Braun-Blanquet cover-abundance scale was applied to describe the ground cover vegetation including species composition and species abundance (Braun-Blanquet 1964). To quantify the biodiversity of the ground vegetation, the Shannon diversity index (H) and Simpson’s dominance index (C_Simpson’s_) were used (Falińska, 1997). On each plot, the tree species were determined and the height and diameter at breast height (1.30 m above ground) of all trees were measured. Based on these data, the total volume (V) of trees (stem and branch volume) was calculated by following equation:

$$ v=\frac{\pi \cdot {d}^2}{40000}\cdot h\cdot f, $$

where: d (cm); h (m); f - form factor from empirical tables for standing trees, that are typically applied in Polish forestry (Czuraj 1991).

### Soil Pollution Indices

The geo-accumulation index (*I*_geo_) and enrichment factor (EF) were calculated to evaluate the degree of pollution in soils formed at the studied spoil heap. The *I*_geo_ index, introduced by Müller (1969), is widely used to assess the heavy metal pollution from anthropogenic sources and is calculated as:

$$ {I}_{\mathrm{geo}}={\mathit{\log}}_2\left[\frac{C}{1.5\ B}\right], $$

where *C* is the content of a measured heavy metal in soil, *B* is the content of a given heavy metal in a bedrock or geochemical background, and the factor 1.5 reflects natural fluctuations of a given heavy metal content in the environment. *I*_geo_ values ≤ 0 represent unpolluted soils, 0–1 represent unpolluted to moderately polluted soils, 1–2 moderately polluted soils, 2–3 moderately to highly polluted soils, 3–4 highly polluted soils, 4–5 represent highly to extremely highly polluted soils, and ≥ 5 extremely polluted soils (Müller 1969).

EF allows assessment of the degree of soil pollution as well as the possible impact of anthropogenic activity on heavy metal content (Kowalska et al. 2016) and is calculated as:

$$ EF=\frac{\left[\left(\frac{C}{Fe\ }\right)\right]\mathrm{sample}}{\left[\left(\frac{C}{Fe\ }\right)\right]\mathrm{background}}, $$

where $\left[\left(\frac{C}{Fe}\right)\right]\mathrm{sample}$ is the ratio of heavy metal to iron content in the soil sample, and $\left[\left(\frac{C}{Fe}\right)\right]\mathrm{background}$ is the ratio of heavy metal to iron content in the geochemical background. EF values < 2 represent minimal soil pollution, 2–5 represent moderately polluted soils, 5–20 represent significantly polluted soils, 20–40 represent highly polluted soils, and > 40 represent extremely polluted soils (Sutherland 2000). Both local as well as reference backgrounds given by Rudnick and Gao (2003) and Kabata-Pendias (2011) were used to calculate background values for *I*_geo_ and EF in this study.

Plant–soil interactions were evaluated by the metal accumulation index (MAI), which enables the assessment of the overall performance of plants in terms of metal accumulation (Liu et al. 2007). MAI is calculated as:

$$ \mathrm{MAI}=\left(\frac{1}{N}\right)\sum \limits_{j=1}^N Ij, $$

where *N* is the total number of analyzed metals, *Ij* = *x* / *δx* is the sub-index for variable *j*, obtained by dividing the mean value (*x*) of each metal by its standard deviation (*δx*) (Liu et al. 2007).

### Statistical Analysis

Distribution maps of individual heavy metal contents in the soil horizon were modeled using the IDW (inverse distance weighted) interpolation method (Isaaks and Mohan Srivastava 1989) using ArcGIS software (Esri, Redlands, CA, USA). To reduce the number of variables in the statistical dataset and to visualize the multivariate dataset as a set of coordinates in a high-dimensional data space, principal components analysis (PCA) was employed.

PCA was also used to interpret other factors, depending on the type of dataset. To confirm the impact of vegetation on the properties of the soil cover encountered on the studied spoil heap, a cluster analysis was conducted that included diverse standardized variables. The biomass volume of trees and Shannon diversity index H were included in the floristic measurements. The selected soil properties subject to examination included the contents of TOC and total N; activities of dehydrogenases, urease, and β-glucosidase; heavy metal content; pH; and particle size. Pearson correlation coefficients between enzyme activity and soil characteristics were also calculated. Multiple regression was used to develop models describing the relationship between the estimated values of enzyme activity and the soil characteristics. All of the statistical analyses were performed using Statistica 10.0 software (StatSoft, Tulsa, OK, USA).

## Results

**Fig. 3:**
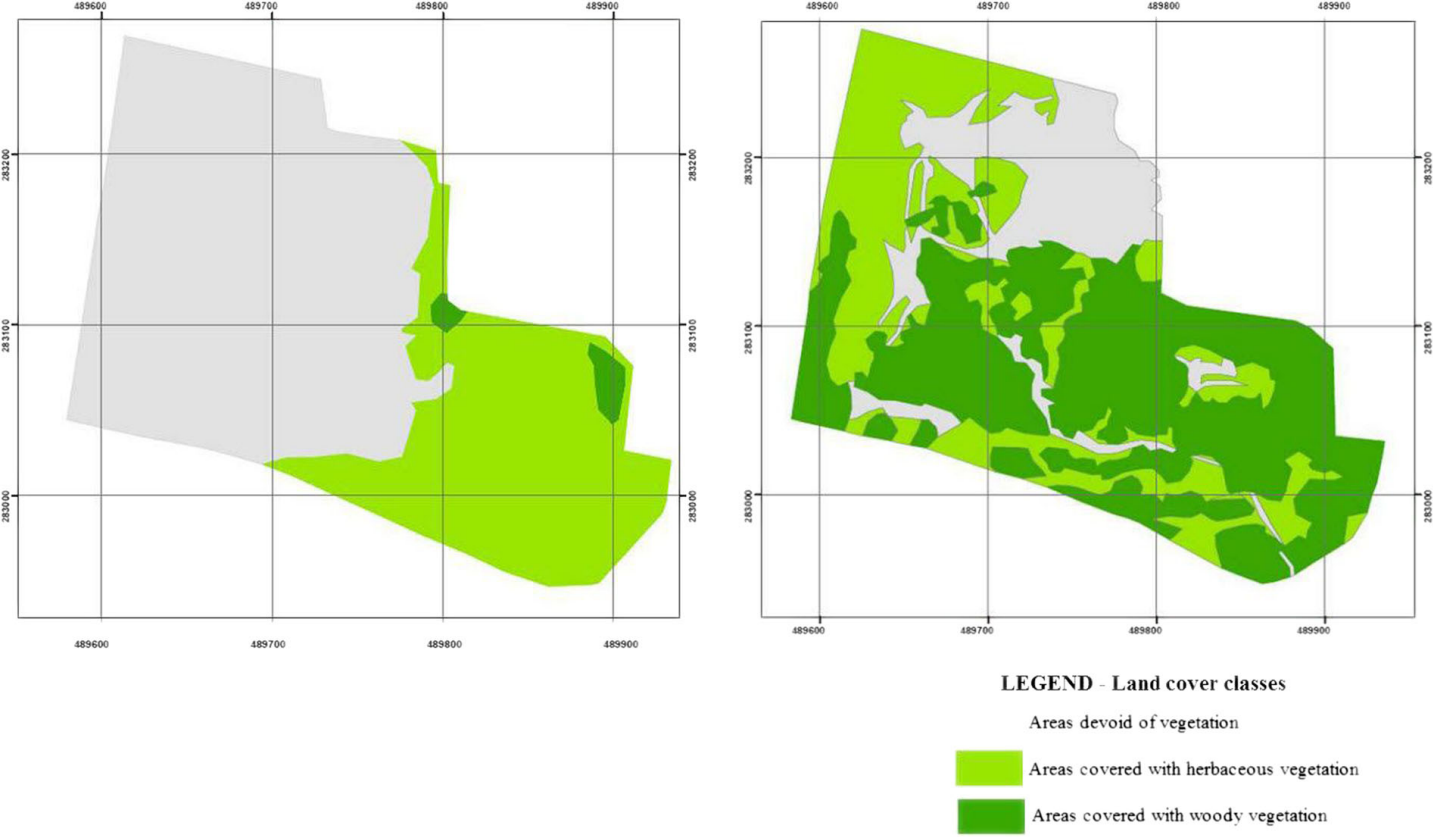
Development of vegetation on sampling area from 1947 (left) to 2011 (right)

Figure 3 shows the changes in the vegetation cover of the spoil heap between 1947 and 2011. In 1947, an area of 2.20 ha (34% of the total heap surface) was covered with plant communities, amongst which herbaceous plant species prevailed (2.11 ha; 33.2% of the total heap surface), while woody species constituted only 0.09 ha (1.5% of the total heap surface). In 2011, the vegetation cover reached 5.15 ha, including woody plants growing on 3.08 ha, and herbaceous plants on 2.17 ha; the amount of spoil heap surface where succession of vegetation was not seen was 3 times smaller than in 1947. The vegetation growing on the heap surface was observed to be diversified in terms of plant species encountered, which was supported by the computed indices of biodiversity (Table 1). The spatial distribution of aboveground plant biomass growing on the spoil heap was highly variable, amounting to 312.4 m^3^ ha^−1^ on average. The highest values of biomass (up to 1470.7 m^3^ ha^−1^) and vegetation biodiversity were recorded in the eastern part of the spoil heap where succession occurred first. In the western part of the heap, the vegetation was the least diversified and more poorly developed.

**Table 1:** Characteristics of the spoil heap vegetation cover (mean ± standard deviation and minimum–maximum values). The study area was separated into three clusters according to the results of PCA analysis

Sampling plots	S	H	C_Simpson’s_	V	Dominant species
First cluster (7 and 9 plot)	62.5 ± 16.3 51–74	1.86 ± 0.19 1.72–2.00	0.035 ± 0.007 0.03–0.04	794.6 ± 956.1118.5–1470.7	*Astragalus glycyphyllos* L., *Betula pendula* ROTH, *Carex brizoides* L., *Carlina vulgaris* L., *Centaurea scabiosa* L., *Chelidonium majus* L., *Elymus repens* (L.) GOULD, *Epipactis atrorubens* (HOFFM.) BESSER, *Eupatorium cannabinum* L., *Euphorbia cyparissias* L., *Festuca ovina* L., *Hieracium pilosella* L., *Leontodon hispidus* L., *Pimpinella saxifraga* L., *Pinus sylvestris* L., *Populus tremula* L., *Rumex acetosa* L.
Second cluster (4,6,8,10–14 plots)	47.9 ± 10.9 31–64	1.75 ± 0.12 1.56–1.99	0.038 ± 0.005 0.03–0.05	344.76 ± 232.17 2.30–571.91	*Arrhenatherum elatius* (L.) P.BEAUV. ex J. PRESL & C. PRESL, *Carex brizoides* L., *Cichorium intybus* L., *Deschampsia caespitosa* (L.) P. BEAUV., *Epipactis helleborine* (L.) CRANTZ, *Festuca ovina* L., *Pimpinella saxifraga* L., *Pinus sylvestris* L., *Ranunculus acris* L. s. s., *Scabiosa ochroleuca* L., *Silene vulgaris* (MOENCH) GARCKE, *Solidago virgaurea* L. s. s.
Third cluster (1–3,5 plots)	33.2 ± 7.9 25–44	1.50 ± 0.15 1.30–1.66	0.048 ± 0.007 0.04–0.06	6.77 ± 12.00 0.00–24.7	*Achillea millefolium* L., *Arrhenatherum elatius* (L.) P.BEAUV. ex J. PRESL & C. PRESL, *Calamagrostis epigeios* (L.) ROTH, *Daucus carota* L., *Echium vulgare* L., *Festuca ovina* L., *Galium mollugo* L., *Lotus corniculatus* L., *Pimpinella saxifraga* L., *Pinus sylvestris* L., *Silene vulgaris* (MOENCH) GARCKE, *Solidago virgaurea* L. s. s., *Thymus pulegioides* L.
All plots	45.8 ± 13.9 25–74	1.7 ± 0.18 1.3–2.0	0.041 ± 0.008 0.03–0.06	312.4 ± 405.6 0.00–1470.7	–

S, species richness; H, Shannon index of diversity; C_Simpson’s_, Simpson’s dominance index; V, volume of trees (stem and branch volume) (m^3^·ha^−1^)

Average values of the physico-chemical soil properties as well as their spatial heterogeneity over the studied spoil heap are shown in Table 2. The texture of the investigated soils was dominated by silt (62–78%), with varying shares of sand (2–28%) and clay (12–23%). The study area was characterized by the most strongly diversified sand fraction (SD = 8.3). The pH_H2O_ and pH_KCl_ of soils ranged from 7.01 to 7.77, and 6.83 to 7.56, respectively. The organic matter content was of particular interest in characterizing the process of soil formation. The variability in total organic carbon (TOC) content was, however high, ranging from 0.77 to 8.01%. The highest TOC content was recorded in soils in the sampling plots 7 and 9, while the lowest in sampling plots 2 and 3. The soil N content ranged between 0.05 and 0.50%. The N content was positively correlated to the TOC content. Base cations ranged from 17.1 to 35.3 cmol(+) kg^−1^.

**Table 2:** The soil characteristics of the spoil heap studied

Plot	pH H_2_O	pH KCl	TOC	N_t_	Ca^2+^	Mg^2+^	Na^+^	K^+^	BC	Sand	Silt	Clay	DH	BG	UR
1	7.68	7.56	1.63	0.05	14.92	2.08	0.01	0.10	17.1	23	63	14	19.11	73.83	0.25
2	7.77	7.54	0.77	0.07	15.94	2.34	0.01	0.17	18.5	22	64	14	32.51	166.25	0.31
3	7.01	6.88	1.17	0.10	16.04	1.76	0.01	0.12	17.9	20	68	12	24.00	155.75	0.23
4	7.52	7.32	1.24	0.09	15.76	1.59	0.01	0.19	17.6	7	75	18	52.50	335.50	0.48
5	7.74	7.51	2.45	0.12	14.87	2.38	0.02	0.17	17.4	28	62	10	53.10	246.58	0.43
6	7.59	7.3	2.20	0.15	15.84	3.42	0.02	0.20	19.5	13	73	14	38.09	225.53	0.55
7	7.22	6.83	8.01	0.50	28.89	5.72	0.03	0.67	35.3	13	74	13	181.37	506.88	1.27
8	7.64	7.35	1.49	0.10	18.93	3.68	0.02	0.36	23.0	2	75	23	43.51	168.17	0.51
9	7.28	6.96	6.14	0.43	25.77	4.66	0.02	0.49	30.9	11	75	14	189.93	519.71	1.51
10	7.53	7.15	2.12	0.13	19.62	4.97	0.02	0.32	24.9	3	76	21	27.44	344.37	0.46
11	7.54	7.28	2.40	0.12	17.15	3.55	0.01	0.21	20.9	8	76	16	46.94	442.90	0.47
12	7.46	7.08	3.39	0.21	19.57	5.29	0.02	0.42	25.3	5	77	18	29.17	500.28	0.62
13	7.63	7.31	2.68	0.15	16.52	4.23	0.02	0.27	21.0	4	78	18	19.48	263.43	0.36
14	7.62	7.28	1.92	0.13	17.72	3.42	0.04	0.31	21.5	5	76	19	28.47	377.52	0.43
Average	7.52	7.24	2.69	0.17	18.40	3.51	0.02	0.29	22.2	12	72	16	56.12	309.05	0.56
SD	0.21	0.23	2.01	0.13	4.14	1.34	0.01	0.16	5.4	8.3	5.3	3.6	56.02	145.88	0.37

SD, standard deviation; TOC, total organic carbon (%); N_t_, total nitrogen (%); Ca^2+^·K^+^·Mg^2+^·Na^+^ (cmol(+) kg^−1^); BC, base cations; sand, silt, and clay (cmol(+) kg^−1^); DH, dehydrogenase activity (μmol TPF·kg^-1^·h^−1^); BG, β-glucosidase activity (mmol pNP·kg^-1^·h^−1^); UR, urease activity (mmol N-NH_4_·kg^-1^·h^−1^)

**Fig. 4:**
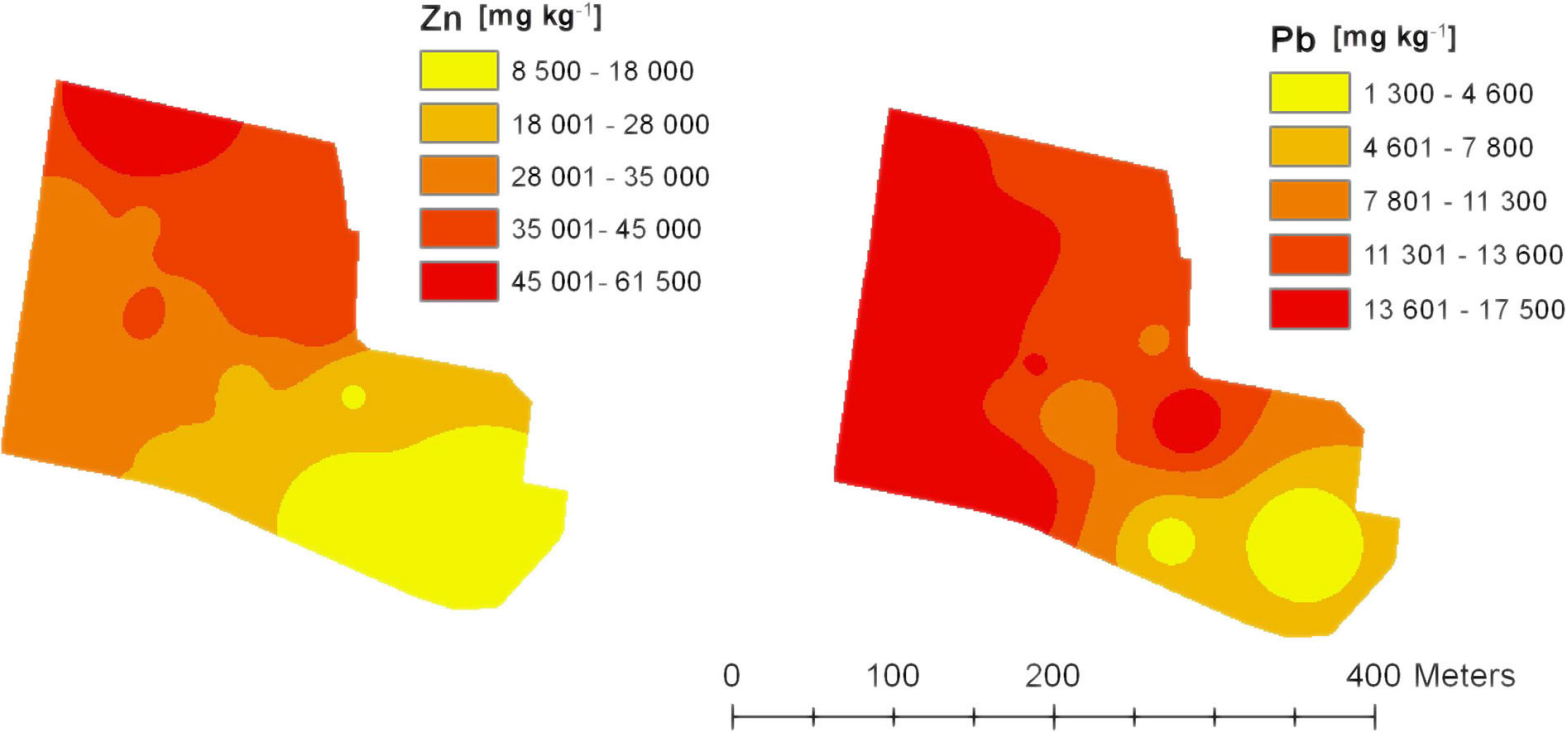
Distribution of Zn and Pb content (mg kg^−1^) in soil in the study area

The studied spoil heap soils had a high spatial variability in terms of their content of heavy metals (Table 3). Extremely high contents of Zn and Pb were recorded. The average Zn content was 31,865 mg kg^−1^ (ranging from 8461 to 61,516 mg kg^−1^). The average Pb content was 12,172 mg kg^−1^ (ranging from 1346 to 17,477 mg kg^−1^). High standard deviations, 14.557 and 4774 for Zn and Pb, respectively, also confirmed the high variability of Zn and Pb content in the soil. The highest contents of both Zn and Pb were recorded in the western and north-western parts of the spoil heap (Fig. 4). The highest contents were seen for Cd and Mn. The average content of Cd was 311.0 mg kg^−1^, and for Mn was 4407 mg kg^−1^. A high content of Fe was recorded, the value of which, depending on the location of the soil sample, accounted for 10.67% (Table 3).

**Table 3:** Heavy metal and Fe content in the soil of study area

Np.	Fe	Zn	Pb	Mn	Cd	Cr	Cu	Ni
1	10.18	30,451	15,012	5401	295.5	153.0	43.4	30.1
2	10.01	30,821	15,697	4381	324.6	82.3	45.8	31.5
3	11.73	61,516	14,402	3738	689.0	133.8	45.6	34.2
4	14.46	47,634	11,377	4122	453.0	153.1	44.6	32.6
5	10.34	44,531	11,047	4479	369.0	89.7	38.0	35.9
6	8.89	17,889	15,777	3970	210.6	108.2	79.2	34.2
7	5.47	8756	3499	3891	88.5	85.9	100.1	39.0
8	13.09	26,294	17,477	5104	243.6	180.3	86.1	47.2
9	4.49	8461	1346	3978	67.0	77.4	50.0	29.1
10	13.72	31,059	13,772	5069	263.5	125.2	67.6	51.9
11	9.87	25,866	8102	3911	246.0	129.1	41.9	34.0
12	14.12	37,029	14,157	4937	368.3	147.6	60.9	49.0
13	13.05	40,991	13,247	5051	362.5	83.8	34.8	47.6
14	9.97	34,814	15,502	3663	372.0	130.9	15.0	39.4
Average	10.67	31,865	12,172	4407	311.0	120.0	53.8	38.3
SD	3.02	14,557	4774	594	153.6	32.5	22.6	7.6

Fe in %; Zn, Pb, Mn, Cd, Cr, Cu, and Ni in mg·kg^−1^

*I*_geo_ values calculated for Zn, Pb, and Cd depended mainly on the applied geochemical backgrounds (Table 4). Nevertheless, *I*_geo_ for these elements were ≥ 5 in all studied soil samples when backgrounds proposed by Kabata Pendias (2011) or Rudnick and Gao (2003) were used, which according to Müller (1969) indicate extremely high pollution. In the case of local background application, for Pb in all soils, *I*_geo_ was ≤ 0 (unpolluted), and for Zn and Cd, *I*_geo_ ranged from ≤ 0 to 1–2 (moderately polluted soils). However, at the same time, in most of the soils, *I*_geo_ for Zn was 0–1 and for Cd ≤ 0. Regardless of whether the local or reference backgrounds were used, *I*_geo_ for Cr and Ni were ≤ 0 or 0–1 with a predominance of unpolluted soils for these elements. The same applied to Cu, but in some plots *I*_geo_ was 1–2 (Table 4).

**Table 4:** The range of *I*_geo_ values and the percentage share of soils according to *I*_geo_ pollution classes

	*I*_geo_	*I*_geo_ pollution classes* [% of soil]						
		≤ 0	0–1	1–2	2–3	3–4	4–5	≥ 5
Local backgrounds								
Zn	−1.46:1.40	22	64	14	–	–	–	–
Pb	−4.31:-0.62	100	–	–	–	–	–	–
Cd	−2.23:1.13	50	43	7	–	–	–	–
Cr	−0.90:0.33	71	29	–	–	–	–	–
Cu	−0.93:1.81	7	57	36	–	–	–	–
Ni	−0.24:0.59	50	50	–	–	–	–	–
Backgrounds by Kabata-Pendias (2011)								
Zn	6.33:9.19	–	–	–	–	–	–	100
Pb	5.90:9.60	–	–	–	–	–	–	100
Cd	8.80:12.17	–	–	–	–	–	–	100
Cr	−0.96:0.27	79	21	–	–	–	–	–
Cu	−2.46:0.28	86	14	–	–	–	–	–
Ni	−0.04:0.79	7	93	–	–	–	–	–
Backgrounds by Rudnick and Gao (2003)								
Zn	6.40:9.26	–	–	–	–	–	–	100
Pb	5.72:9.42	–	–	–	–	–	–	100
Cd	8.96:12.32	–	–	–	–	–	–	100
Cr	−0.84:0.39	71	29	–	–	–	–	–
Cu	−1.49:1.25	22	64	14	–	–	–	–
Ni	−1.28:-0.44	100	–	–	–	–	–	–

*Interpretation of *I*_geo_ pollution classes is given in pollution indices chapter

Similar to the case of *I*_geo_, the values of *EF* for Zn, Pb, and Cd were strongly related to the geochemical background applied (Table 5). Applying the local backgrounds, *EF* for these metals was in most soils < 2 (minimal soil pollution). A much greater diversity of *EF* values appeared if backgrounds proposed by Kabata-Pendias (2011) or especially Rudnick and Gao (2003) were used. On the basis of the background of Kabata-Pendias (2011), almost all studied soils were significantly or highly polluted with Zn, Pb, and Cd (*EF* 5 to 40). The background of Rudnick and Gao (2003) indicated a lower degree of pollution. In this case, *EF* values calculated for Zn and Pb were < 2 to 2–5 (moderate soil pollution), but for the majority of the soils the *EF* for Cd was 20–40. Using reference backgrounds did not indicate pollution of studied soils with Cr, Cu, and Ni. When local backgrounds were used, the majority of studied spoil heap soils were minimally polluted with Cr and Ni and moderately polluted with Cu (Table 5).

**Table 5:** The range of EF values and the percentage share of soils according to EF pollution classes

	EF	EF pollution classes* [% of soil]				
		< 2	2–5	5–20	20–40	> 40
Local backgrounds						
Zn	0.84:2.74	86	14	–	–	–
Pb	0.14:−0.77	100	–	–	–	–
Cd	0.30:3.28	93	7	–	–	–
Cr	0.42:2.52	86	14	–	–	–
Cu	0.58:5.86	36	57	7	–	–
Ni	0.32:2.18	93	7	–	–	–
Backgrounds by Kabata-Pendias (2011)						
Zn	11.43:37.46	–	–	50	50	–
Pb	5.55:32.87	–	–	57	43	–
Cd	18.20:71.63	–	–	15	64	21
Cr	0.05:0.14	100	–	–	–	–
Cu	0.02:0.24	100	–	–	–	–
Ni	0.04:0.12	100	–	–	–	–
Backgrounds by Rudnick and Gao (2003)						
Zn	1.48:4.85	29	71	–	–	–
Pb	1.09:6.46	7	93	–	–	–
Cd	10.26:40.40	–	–	64	29	7
Cr	0.00:0.01	100	–	–	–	–
Cu	0.00:0.04	100	–	–	–	–
Ni	0.00:0.01	100	–	–	–	–

*Interpretation of EF pollution classes is given in pollution indices chapter

The calculated MAI index of Scots pine (2.52) was slightly higher than *S. vulgaris* (1.63). The average contents of Zn, Pb, and Cd in the aboveground biomass of *S. vulgaris* were 2-, 5-, and 5-times higher, respectively, when compared with those in the second-generation needles of Scots pine (Table 6). The average content of Zn in the pine needles was 212.3 mg kg^−1^ but was 402.9 mg kg^−1^ in *S. vulgaris*. Similarly, the average content of Pb in pine needles was 14.3 mg kg^−1^ but was 70.1 mg kg^−1^ in *S. vulgaris*. In the case of Cd, Cr, and Ni, a higher accumulation capacity was also seen in *S. vulgaris* (Table 6). The content of Zn in pine needles positively correlated with the contents of Zn and Cd in the soil. The Zn content in *S. vulgaris* biomass correlated positively with contents of Zn, Pb, Fe, and Cd in the soil, while it negatively correlated with soil organic carbon, N and Ca content (Table 7). The amount of Pb in the aboveground biomass of *S. vulgaris* revealed a positive correlation with the content of Pb within the soil (Table 7).

**Table 6:** Heavy metal content in Scots pine needles and *S. vulgaris* aboveground biomass

	Zn	Pb	Mn	Cd	Cr	Cu	Ni
*Pinus sylvestris* – needles							
Average	212.3	14.3	110.2	1.1	3.5	2.9	0.1
Range	150.4–323.5	6.6–33.0	50.7–1778.7	0.5–1.6	1.3–6.6	0.8–4.7	0.0–0.3
SD	51.9	8.6	40.6	0.4	1.3	1.2	0.1
*Silene vulgaris* – aboveground biomass							
Average	402.9	70.1	34.3	5.7	5.3	2.1	3.0
Range	74.6–736.5	5.6–198.9	9.3–70.2	1.0–11.0	0.0–14.5	0.0–5.2	0.0–6.5
SD	191.6	50.2	15.4	2.9	5.2	1.4	2.3

SD, standard deviation; Zn, Pb, Mn, Cd, Cr, Cu, and Ni in mg·kg^−1^

**Table 7:** Pearson correlation coefficient between heavy metal content in Scots pine needles and *S. vulgaris* aboveground biomass and soil characteristics

	C	N	Fe	Ca	Zn	Pb	Cd
Pine needles							
Zn	−0.48	−0.34	0.52	−0.31	0.58*	0.41	0.60*
Pb	−0.01	−0.07	0.05	−0.22	0.30	−0.02	0.27
*Silene vulgaris*							
Zn	−0.58*	−0.62*	0.79*	−0.72*	0.77*	0.69*	0.74*
Pb	−0.41	−0.51	0.52	−0.43	0.43	0.68*	0.46

*P < 0.05

The biological activity of the studied soils, expressed in terms of activity of soil enzymes, had a high spatial variability (Table 2). Dehydrogenase activity varied from 19.11 to 189.93 μmol TPF kg^−1^ h^−1^. The average activity of β-glucosidase amounted to 309.5 mmol pNP kg^−1^ h^−1^, while the highest detected activity exceeded 500 μmol TPF kg^−1^ h^−1^. The activity of urease ranged between 0.23 and 1.51 mmol N-NH_4_ kg^−1^ h^−1^. The highest activities of all studied enzymes were recorded in the eastern part of the spoil heap (sampling plots 7 and 9), whereas the lowest activities were found in the western part (plots 1 and 3). Multiple regression models explained 61% to 90% of the variance in enzyme activity (Table 8). DH activity was largely dependent on Pb content; multiple regression revealed that Pb content accounted for 77% of the variation in the DH activity. Amongst the tested soil parameters, the contents of N and Cd explained 90% of the UR activity variance. About 61% of the variance in the BG activity was explained by the N content (Table 8).

**Table 8:** Multiple regression analysis for enzymes activity based on soil characteristics. *R*^2^ describes the percentage of explained variance. *β* is the regression coefficient for given equation parameter and *p* is the significance level for the equation parameter

Enzyme activity	*R* ^2^	Equation parameter	*β*	*p*
DH	77%	logPb	−0.874	0.0001
UR	90%	logN	0.488	0.0015
		logCd	−0.379	0.0087
BG	61%	logN	0.709	0.0009

DH, dehydrogenase activity; UR, urease activity; BG, β-glucosidase activity

**Fig. 5:**
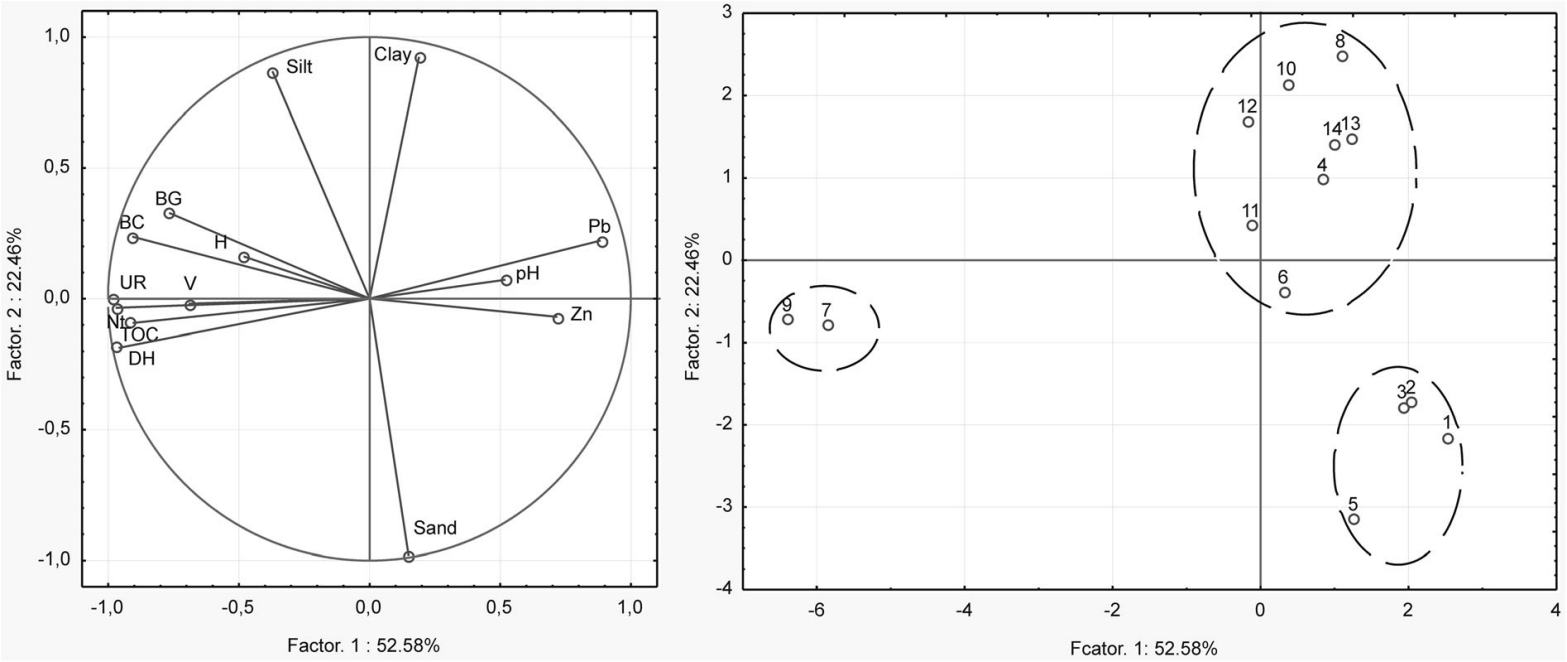
The projection of measured variables on a plane of the first and second PCA factors

The PCA revealed that the first principal component (Factor 1) explained approximately 53% of the dataset variation (Fig. 5) and was primarily related to the activity of soil enzymes and heavy metal contents in the spoil heap. These variables were strongly negatively correlated. The second principal component (Factor 2) explained about 22% of the variation and referred mainly to the soil texture. The distribution of cases on the plane of Factors 1 and 2 confirms the regularities discussed above.

To confirm the impact of vegetation on the properties of the soil cover encountered on the studied spoil heap, a cluster analysis was conducted, which included diverse standardized variables. The biomass volume of trees and Shannon diversity index H were included in the floristic measurements. The selected soil properties included were the contents of organic carbon and nitrogen, activities of dehydrogenases, urease and β-glucosidase, heavy metal content, pH, and particle size. The cluster analysis based on the abovementioned properties indicated an existence of three clusters, to a large extent overlapping with the clusters visible in Fig. 5, presenting the projection of cases on the factor plane in the PCA. The first cluster corresponded to the spoil heap plots with low heavy metal content, high values of the vegetation biomass and activity of soil enzymes, and relatively high content of soil organic matter and nitrogen. The second cluster showed the plots with the highest heavy metal contents, while the third cluster included the plots with a prevailing sand fraction (Fig. 5).

The distinguished clusters varied in terms of their plant species biodiversity. The first cluster had the highest abundance of vegetation with an average *S* value of 62.5, while average *S* values of 47.9 and 33.2 were found for the spoil heap plots in the second and third clusters, respectively. The first cluster was also characterized by the highest value of Shannon diversity index (1.66 on average), while it was only 1.75 and 1.50 for the second and third clusters, respectively. Similar dependences were revealed in respect to the average volume of the aboveground biomass (Table 1).

## Discussion

The content of heavy metals determined from spoil heap soils under study exceeded the admissible standards, especially for Zn, Cd, and Pb. According to Kabata-Pendias (2011), the acceptable thresholds of heavy metal contents are 1 mg kg^−1^ for Cd, 30 mg kg^−1^ for Cu, 20 mg kg^−1^ for Ni, 50 mg kg^−1^ for Cr, 50 mg kg^−1^ for Pb, and 100 mg kg^−1^ for Zn. Pb, Zn, and Cd contents in soils were 250–300 times above the threshold values, while Cu, Ni, and Cr contents were only 2–2.5 times above the threshold values. This was also confirmed by the calculated soil pollution indices, *I*_geo_ and *EF*, especially in the case of *I*_geo_ when backgrounds proposed by Kabata-Pendias (2011) were used. However, the application of local backgrounds (i.e., spoil heap deposits) indicated only slight contamination. The high values of *I*_geo_ and *EF* indicators, when using the reference background clearly indicate the anthropogenic origin of the deposited material of the heap (Kowalska et al. 2016). Higher exceedances of allowed limits for contents of heavy metals in soils were recorded in the western part of the spoil heap, where the biomass volume and biodiversity of plant species were also the smallest.

The quantity and spatial distribution of heavy metals within the soil affected the natural succession of vegetation. According to Woch et al. (2016), an increased content of heavy metals might be a dominant factor in determining the distribution of particular plant species. Numerous studies have shown a tight relationship between the abundance and biodiversity of plant species and the occurrence of certain heavy metals (Becker and Brändel 2007; Pandey et al. 2015). Here we found that contents of Zn, Pb, and particularly Cd in soils of the spoil heap determine the composition of the vegetation cover. The number of plant species decreased with increasing soil contamination by heavy metals. Moreover, the numbers of species susceptible to these metals was smaller, and the species more tolerant to soil contamination prevailed (Table 1). Woch et al. (2016), upon investigating sites strongly polluted with heavy metals, reported that the vegetation encountered in such environments was dominated by species with wide ecological amplitude and small competitiveness, for example *S. vulgaris*. This observation was confirmed here, since this was the predominant species on the studied spoil heap. Our study also revealed substantially higher accumulation capacity for heavy metals in *S. vulgaris* leaves and stems than that of Scots pine needles. The low MAI of *S. vulgaris* resulted from the high standard deviations of the metals analyzed here and microhabitat variation. The calculated MAI value for both species analyzed here were very similar to those in the studies of Monfared et al. (2013), where they were 1.9 to 2.4 for *Plantanus orientalis*, *Robinia pseudoacacia*, and *Fraxinus rotundifolia* and much lower than in the studies of Liu et al. (2007), where they were 53.8 for *Catalpa speciosa* leaves. In order to properly confirm the suitability of plant, and particularly tree, species in phytoremediation of areas contaminated with heavy metals, it would be appropriate to calculate also other indicators such as BCF, BAF, and/or TF (Heinonen et al. 1999, Tudor et al. 2006, Rana and Maiti 2018).

A high spatial heterogeneity in activities of soil enzymes was found to correlate negatively with the content of heavy metals in the spoil heap soil. Ciarkowska et al. (2014) demonstrated that heavy pollution with Zn, Pb, and Cd slightly inhibited enzyme activity. The highest activity of enzymes within the investigated spoil heap was recorded in its eastern part, where the content of Zn and Pb constituted 30% of the average content of these elements in the soils of the spoil heap.

To identify soil properties stimulating the activity of the enzymes under analysis multiple regression was used. Multiple regression models explained from 61% to 90% of the variance in the enzyme activity. The DH and UR were largely dependent on the heavy metal content within the soil, the Pb and Cd contents in particular. The activity of β-glucosidase was only little explained by such models, and the model did not include any heavy metals. The results obtained might indicate that the activity of dehydrogenases and urease is more suitable for assessing the changes in the soils strongly polluted with heavy metals compared to β-glucosidase. Wyszkowska et al. (2010) reported that the contamination of soil with copper significantly inhibited its biochemical activity, while β-glucosidase appeared to be the least susceptible to the occurrence of this element.

PCA confirmed a strong, positive correlation of soil enzyme activity with the biomass production and biodiversity of vegetation covering the spoil heap, as well as with the content of soil organic matter. The vegetation affects the quantity and quality of the soil organic matter (Gruba et al. 2015; Błońska et al. 2016a), and the latter determines biological processes that develop within the soil. According to Singh et al. (2001) and Harris (2003), along with the accumulation of organic carbon in soils, which are subject to restoration processes influenced by different disturbing factors, the microbial biomass carbon increases in time, stimulating the changes that occur in the microbial structure; these authors conclude measurements of the soil microbial community constitutes a fine tool for the assessment of development and the level of soil restoration.

Earlier studies have shown that Zn contents in plant tissues above 400 mg kg^−1^ (Alloway 1990) or even 100 mg kg^−1^ (Kabata-Pendias 2011) are toxic for plants. Similarly, Pb content in plant leaves between 30 and 300 mg kg^−1^ is considered to be phytotoxic (Kabata-Pendias and Pendias 2001; Padmavathiamma and Li 2007). High contents of Zn and Pb found in the aboveground biomass of *S. vulgaris* (Table 6) thus support earlier studies (Verkleij and Prast 1989; Ernst 2006), which considered *S. vulgaris* to be a facultative metallophyte. In our study, the average contents of Zn, Pb, and Cd in the aboveground biomass of *S. vulgaris* were ca. two, five, and five times higher, respectively, when compared with the content of these elements in the second-generation needles of Scots pine*.* Scots pine is often used as a bio-indicator to detect environmental pollution (Greszta et al. 2002; Pająk et al. 2017). Such a high accumulation of heavy metals in the aboveground parts of *S. vulgaris* indicates that they can be potentially used in phytoremediation of post-industrial sites.

In respect to pine needles, Kabata-Pendias and Piotrowska (1984) reported that the naturally occurring content of Zn ranges between 10 and 100 mg kg^−1^, while Greszta and Panek (1989) postulated a range of 15–80 mg kg^−1^. The content of Zn in needles of pines growing on the heap studied here was 202.12 mg kg^−1^. Kabata-Pendias and Piotrowska (1984) established that a normal content of Cd in needles reaches the value of 1 mg·kg^−1^, and up to 30 mg·kg^−1^ for Pb. The average contents of these elements within needles of trees growing on the spoil heap remained within the abovementioned ranges.

## Conclusions

The results obtained in this study confirmed the presence of a high degree of pollution in the spoil heap soils. The heavy metal content determined exceeded the acceptable thresholds. The results indicated that heavy metal pollution had a significant impact on the soil biochemical activity and vegetation development. Heavy metal pollution particularly reduced activities of dehydrogenases and urease. Enzyme activity negatively correlated especially with the content of Pb and Cd in the soil. In addition, the soil organic matter content significantly determined the biochemical activity of soils. Vegetation affected the quantity and quality of soil organic matter, which determined the biochemical processes ongoing within the soil. The greater usefulness of dehydrogenases and urease activity in assessing the quality of soils strongly contaminated with heavy metals was shown. The high accumulation of Zn, Pb, and Cd in the aboveground parts of *S. vulgaris* indicated an unusual tolerance for high heavy metal concentration in soil and possibility of using this species in phytoremediation of post-industrial sites.
